# Arabinoxylan Microspheres: Structural and Textural Characteristics

**DOI:** 10.3390/molecules18044640

**Published:** 2013-04-19

**Authors:** Ana L. Martínez-López, Elizabeth Carvajal-Millan, Mario Miki-Yoshida, Lorena Alvarez-Contreras, Agustín Rascón-Chu, Jaime Lizardi-Mendoza, Yolanda López-Franco

**Affiliations:** 1 Laboratory of Biopolymers, CTAOA. Research Center for Food and Development, CIAD, A.C. Carretera a La Victoria Km. 0.6, Hermosillo, Sonora, 83000 Mexico; E-Mails: ana.martinez@estudiantes.ciad.mx (A.L.M.-L.); jalim@ciad.mx (J.L.-M.); lopezf@ciad.mx (Y.L.-F.); 2 Centro de Investigación en Materiales Avanzados S.C. Miguel de Cervantes 120, Chihuahua, Chih. CP 31109, Mexico; E-Mails: mario.miki@cimav.edu.mx (M.M.-Y.); lorena.alvarez@cimav.edu.mx (L.A.-C.); 3 Laboratory of Biotechnology, CTAOV. Research Center for Food and Development, CIAD, A.C. Carretera a La Victoria Km. 0.6, Hermosillo, Sonora, 83000 Mexico; E-Mail: arascon@ciad.mx

**Keywords:** maize bran arabinoxylans, microspheres, ferulic acid, microstructure

## Abstract

The aim of this research was to study the structural and textural characteristics of maize bran arabinoxylan (MBAX) microspheres. The laccase-induced cross-linking process was monitored by storage (G') and loss (G'') moduli changes in a 4% (w/v) MBAX solution. The G' and G'' values at the plateau region were 215 and 4 Pa, respectively. After gelation, the content of ferulic acid dimers decreased from 0.135 to 0.03 µg/mg MBAX, suggesting the formation of ferulated structures unreleased by mild alkaline hydrolysis. MBAX microspheres presented an average diameter of 531 µm and a swelling ratio value (*q*) of 18 g water/g MBAX. The structural parameters of MBAX microspheres were calculated from equilibrium swelling experiments, presenting an average mesh size of 52 nm. Microstructure and textural properties of dried MBAX microspheres were studied by scanning electron microscopy and nitrogen adsorption/desorption isotherms, respectively, showing a heterogeneous mesoporous and macroporous structure throughout the network.

## 1. Introduction

Microencapsulation of bioactive agents has recently become a relevant alternative to develop novel oral delivery systems. Microspheres can encapsulate many types of drugs, including small molecules, proteins and nucleic acids, thus providing improved oral bioavailability [1]. While a variety of devices have been used as a carrier material for bioactive agents, biopolymer-based microspheres offer several advantages. Due to their attractive biodegradable, biocompatible, non-toxic and hydrophilic properties and their mild cross-linking conditions, natural polysaccharides such as chitosan, alginate, dextran, among others, have received increasing attention as oral delivery systems [2,3,4,5,6,7]. Hydrogel polysaccharides, particularly in the form of microspheres, have been extremely useful in the controlled release of bioactive agents and their targeting to selective sites [8,9].

**Figure 1:**
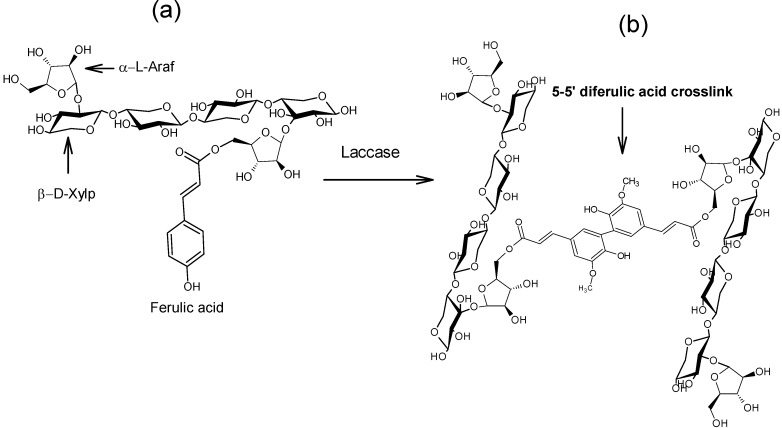
Schematic representation of the covalent cross-linking of ferulated arabinoxylans. Ferulated arabinoxylans (**a**) in solution and (**b**) in gel. Covalent cross-linking through a 5,5 diferuloyl moiety in the gel is presented as an example. α-L-Araf = α-L-arabinofuranose, β-D-Xylp = β-D-xylopyranose.

Hydrogels are three-dimensional polymer networks capable of imbibing large amounts of water [9]. Covalently cross-linked gels generally present high water absorption capacity, absence of pH or electrolyte susceptibility and exhibit no syneresis after long periods of storage [10]. An example of covalently cross-linked gels is the product of oxidative coupling of ferulated arabinoxylan chains. Arabinoxylans (AX) are important cereal non-starch polysaccharides constituted of a linear backbone of β-(1→4)-linked D-xylopyranosyl units to which α-L arabinofuranosyl substituents are attached through O-2 and/or O-3 [10]. Some of the arabinose residues are ester-linked on (O)-5 to ferulic acid (FA, 3-methoxy-4 hydroxycinnamic acid) [11]. One of the most important properties of AX is the ability to form gels by covalent cross-linking involving FA oxidation by either chemical (ferric chloride, ammonium persulphate) or enzymatic (peroxidase/H_2_O_2_, laccase/O_2_) free radical-generating agents [10,12,13,14,15]. This oxidation allows the coupling of AX chains through the formation of dimers and trimers of FA (di-FA, tri-FA), generating an aqueous three-dimensional network (Figure 1). The content of covalent bonds in the gel is determined by the extent of oxidative coupling of FA and can be quantified by the formation of di-FA and tri-FA [16]. Five isomeric forms of di-FA structures have been reported in AX gels: 5-5'-, 8-5'-benzo-, 8-*O*-4'-, 8-5'- and 8-8'- [17,18] and only one tri-FA: 4-*O*-8', 5-5'- [19]. In addition to covalent bonds (di-FA and tri-FA), physical interactions between AX chains can contribute to the gelation process [12,16]. 

Previous studies have demonstrated that AX gels formed via the oxidative cross-linking using laccase could be employed for controlled release of model proteins [17,19,20], methyl xanthine [21] and lycopene [22], making AX hydrogels good candidates for the design of novel colon-specific delivery systems. Recently, the formation of laccase-induced AX microspheres stabilized by covalent linkages has been reported for the first time [23]. AX microspheres present several advantages compared with other polysaccharide microspheres as covalently cross-linked networks are strong, form quickly, are stable upon heating and exhibit no syneresis after extended storage [10]. However, the structural and textural characteristics of AX microspheres have not been reported yet. In this study, maize bran arabinoxylans (MBAX) presenting gelling capability were used to prepare MBAX microspheres which were then investigated in terms of their structural and textural characteristics.

## 2. Results and Discussion

### 2.1. MBAX Cross-Linking

**Figure 2:**
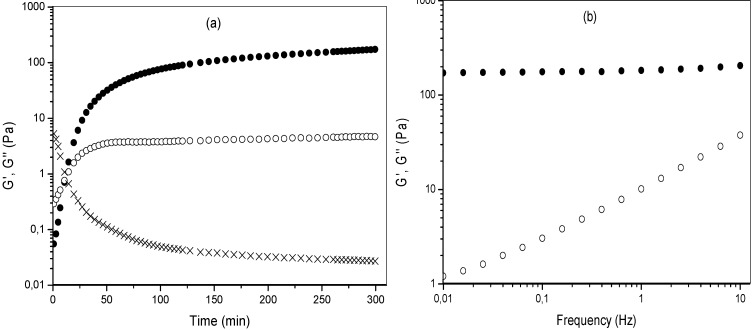
(**a**) Monitoring the tan δ (X), storage (G'●) and loss (G''❍) modulus of MBAX solution during gelation by laccase at 25 °C, 0.25 Hz and 5% strain; (**b**) Mechanical spectrum of MBAX gels at 6 h (G'●, G''❍). Data obtained at 25 °C and 5% strain.

The MBAX gelation was monitored by storage (G') and loss (G'') moduli changes in a 4% (w/v) polysaccharide solution undergoing oxidative gelation by laccase (Figure 2a). The gelation profile *vs.* time exhibited an initial increase of G' modulus, followed by a plateau region. The rheological measurements indicated that the gelation time (tg), calculated from the crossover of the G' and G" curves (G' > G") was 4 min. The tg value indicates the sol/gel transition point and at this point G' = G" or tan δ = G''/G' = 1 [24]. The values of G' and G'' at the plateau region were 215 and 4 Pa, respectively. The tan δ value decreased during MBAX gelation (Figure 2a) indicating the presence of an elastic covalent system. The mechanical spectra of the MBAX gel obtained for 6 h gelation (Figure 2b), was typical of a solid-like material with a linear G' independent of frequency and G" much smaller than G" and dependent of frequency. This frequency-independent behavior is indicative of a stable, cross-linked network and similar to that reported for AX gels [14,25]. The high G’ value of MBAX gel has been attributed to the covalent cross-linking content and to the physical entanglement of MBAX chains [17]. The extent of covalent cross-linking in MBAX gels was determined by the content of ferulate monomer and total di-FA and tri-FA before and after 6 h of gelation (Table 1). FA was oxidized (82% of initial FA content) during the gelation process. After gelation, the di-FA content in MBAX gels did not increase, but rather decreased from 0.135 to 0.03 µg/mg MBAX. The tri-FA was present only in trace quantities (0.003 µg/mg MBAX). Nevertheless, the tan δ (G''/G') values confirm the formation of a true gel after laccase treatment (Figure 2b). This behavior in MBAX gels has been previously reported by several authors [14,15,24,25,26]. These authors attributed this result to the formation of ferulated cross-linking structures which cannot be released by mild alkaline hydrolysis and/or to the participation of lignin residues in the formation of MBAX gel. The relative percentages of each di-FA structures in MBAX gels were: 78%, 14% and 8% for the 8-5' (mainly in the benzofuran form), 5-5' and 8-*O*-4' structure, respectively. Previous research on MBAX gels induced by peroxidase/H_2_O_2_ system also reported the 8-5' di-FA structure as predominant [14].

### 2.2. MBAX Microspheres

**Figure 3:**
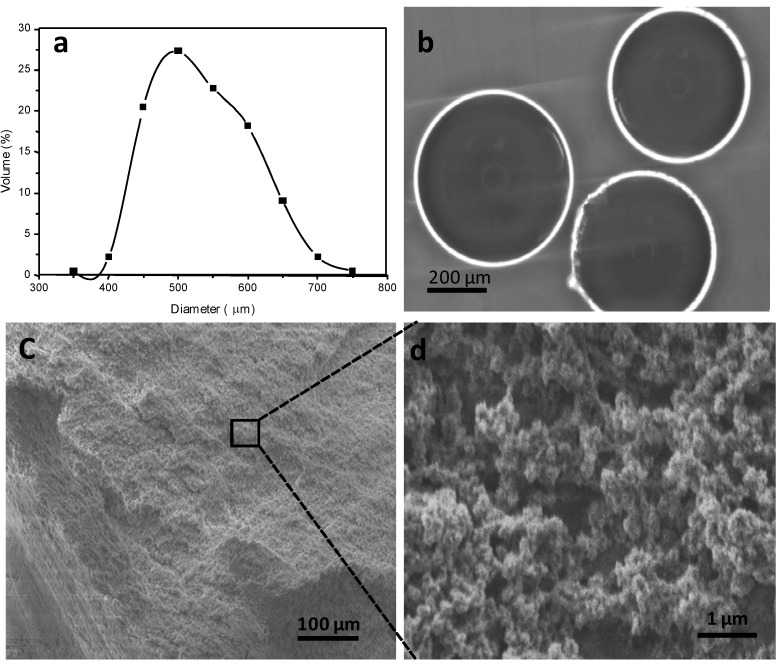
Characterization of MBAX microspheres. (**a**) Diameter distribution; (**b**) Optical microscope observation; (**c** and **d**) SEM images.

The MBAX microspheres presented a size distribution from 350 µm up to 798 µm, with an average diameter of 531 µm (Figure 3a). These particle size values were similar to those reported for other polysaccharide microspheres such as alginate (350 µm) [27]. Optical micrographs of MBAX microspheres show a spherical shape and no aggregation of particles (Figure 3b). Secondary electron SEM images of cross-sectional MBAX microspheres is shown in Figure 3c,d. The microsphere morphology presented a three-dimensional and heterogeneous network structure, with irregular pores size and geometries (Figure 3c). The SEM micrograph of Figure 3d (magnification), shows clusters of interconnected nodular structures. The aggregation of nodular structures in clusters allows pore sizes of 10–70 nm, while the binding with other clusters leads to pores sizes on the macroporous order. It has been reported that for gels cross-linked via phenolic groups, the pore size is determined by the presence of nodular clusters [28,29,30,31,32,33]. Thus, the heterogeneous microstructure of MBAX microspheres could be attributed to the content and distribution of ferulic acid covalent cross-linking structures forming the MBAX network.

The swelling behavior of MBAX microspheres was followed for 30 min at 25 °C, with the equilibrium being reached between 8–12 min. This water dissolution mechanism of the MBAX microspheres was fitted to the Fick model. The n value was 0.19, indicating that the dissolution is due to a Fickian diffusion, where water penetration rate in the microsphere is lower than the polymer chain relaxation rate. The swelling ratio value (*q*) in MBAX microspheres was 18 g water/g MBAX, which is similar to the value reported for MBAX hydrogels (20 g water/g AX) [24]. Nevertheless, MBAX microspheres showed a very short swelling time to reach equilibrium (12 min) compared to that reported for cylindrical MBAX hydrogels (15 h) [24]. This behavior could be explained in terms of an increase in the surface area-to-volume ratio in MBAX microspheres. 

The molecular weight between two cross-links (Mc), the cross-linking density (ρc) and the mesh size (*ξ*) values of gelled MBAX are presented in Table 1. Similar structural parameters have been reported for maize bran and wheat bran arabinoxylan gels [14,34]. Higher mesh sizes values (201–331 nm) have been reported in laccase-induced wheat flour AX gels at lower polysaccharide concentrations (0.5%–2% w/v) [17] and higher di-FA and tri-FA contents. The latter could be related to the high molecular weight reported for arabinoxylans from wheat flour (438 kDa) [17] in comparison to the alkali-extracted arabinoxylans from maize bran (197 kDa) used in the present study [35]. 

**Table 1:** Characteristics of MBAX before and after 6 h of cross-linking process.

**Characteristic**	**t = 0 h**	**t = 6 h**
FA (μg/mg MBAX)	0.255 ± 0.017	0.045 ± 0.002
di-FA (μg/mg MBAX)	0.135 ± 0.011	0.03 ± 0.001
tri-FA (μg/mg MBAX)	0.064 ± 0.010	Traces
Mc ^a^ ×10 ^3^ (g/mol)	-	24 ± 0.4
*ρ*_c_^b^ × 10^−6^ (mol/cm^3^)	-	59 ± 0.2
*ξ* ^c^ (nm)	-	52 ± 1.1

^a^ Molecular weight between two cross-links; ^b^ Cross-linking density; ^c^ Mesh size; All values are means ± standard deviation of three repetitions.

**Figure 4:**
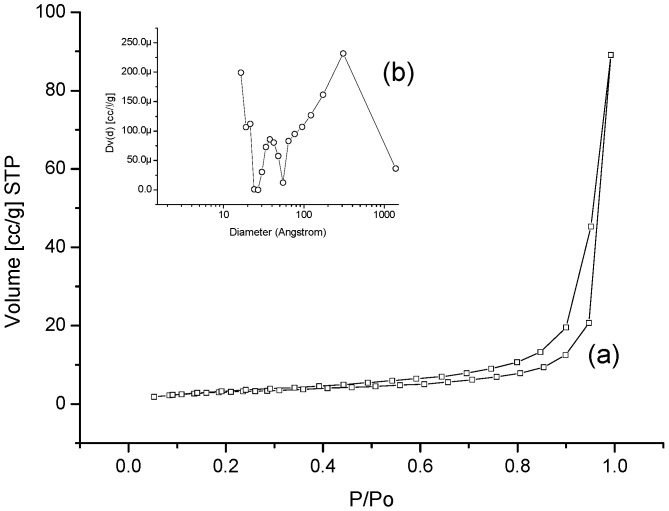
Textural analysis of MBAX microspheres. (**a**) Isotherm; (**b**) Pore size distribution.

The nitrogen adsorption/desorption isotherm of the MBAX microspheres is presented in Figure 4a. The N_2_ adsorption measurements indicate that the specific surface area of MBAX microspheres is 11.19 m^2^/g, beside; the isotherm was type IV, denoting the presence of mesopores [36]. Furthermore, a broad hysteresis loop associated with a desorption step above the relative pressure of 0.5 shown in N_2_-isotherm is characteristic of a mesoporous organization. The calculation of pore size distribution in MBAX microspheres was based on the BJH method using the desorption isotherms (Figure 4b). The average pore size in microspheres was 30.8 nm, a value consistent with the mesoporous structure calculated from swelling experiments (Table 1). However, it should be emphasized that the absence of a plateau at high pressures in the isotherms and a non-Gaussian pore diameter distribution (Figure 4a,b) indicates a macroporous material. Nevertheless, it could also be attributed to apparent porosity, which could produce macro-cavities. These results confirm the heterogeneous microtexture observed in the SEM micrographs.

## 3. Experimental

### 3.1. Materials

Maize bran arabinoxylans (MBAX) were obtained and characterized as previously reported [35]. MBAX contain 85% dry basis (d.b.) of pure AX. MBAX presented a ferulic acid (FA), di-FA, and tri-FA content of 0.25, 0.14, and 0.07 μg/mg of MBAX, respectively, and an A/X ratio of 0.72. Laccase (benzenediol: oxygen oxidoreductase, E.C.1.10.3.2) from *Trametes versicolor* and all other chemical products were purchased from Sigma Chemical Co. (St. Louis, MO, USA).

### 3.2. Methods

#### 3.2.1. MBAX Gelation

The reaction mixtures contained MBAX solution at 4% (w/v) in 0.1 M acetate buffer pH 5, laccase (1.670 nKat/mg of MBAX). 

##### 3.2.1.1. Rheological Tests

The formation of the MBAX gel was followed using a strain-controlled rheometer (Discovery HR-3 rheometer, TA Instruments, New Castle, DE, USA) in oscillatory mode as follows: cold (4 °C) solutions of 4% (w/v) MBAX were mixed with laccase and immediately placed in the cone and plate geometry (5.0 cm in diameter, 0.04 rad in cone angle) maintained at 4 °C. MBAX gelation kinetic was started by a sudden increase in temperature from 4 to 25 °C and monitored at 25 °C for 6 h by following the storage (G') and loss (G'') modulus and tan δ (G''/G'). All measurements were carried out at a frequency of 0.25 Hz and 5% strain (linearity range of visco-elastic behavior). Frequency sweep (0.01–10 Hz) was carried out at the end of the network formation at 5% strain and 25 °C.

##### 3.2.1.2. Covalent Cross-Links Content

FA, di-FA and tri-FA contents in MBAX microspheres were quantified by reverse phase high-performance liquid chromatography (RP-HPLC) after a deesterification step, as described elsewhere [12,18]. An Alltima (Alltech, Deerfield, IL, USA) C18 column (250 × 4.6 mm) and a photodiode array detector Waters 996 (Millipore Co., Milford, MA, USA) were used to record the ferulic acid and its di-FA and tri-FA spectra. Detection was by UV absorbance at 320 nm. Gradient elution was performed using acetonitrile and sodium acetate buffer 0.05 M, pH 4.0, at 1 mL/min at 35 °C, in linear gradients from 15:85 to 35:65 in 30 min, from 35:65 to 60:40 in 0.5 min, from 60:40 to 15:85 in 4.5 min, and finally maintained at 15:85 for 5 min.

#### 3.2.2. MBAX Microspheres

MBAX microspheres were prepared as previously described [23]. Briefly, the MBAX solution 4% (w/v) in 0.1 M acetate buffer pH 5 was mixed with 1.670 nKat/mg of MBAX. This reaction mixture was transferred to a syringe and equilibrated for approximately 4 min. Microspheres were obtained by dropwise extrusion into a hydrophobic liquid. The microspheres were aged for 12 h in this liquid at 25 °C under stirring. Afterwards MBAX microspheres were collected and washed with water–ethanol (1:1) in order to remove the residual hydrophobic liquid from the microsphere surface and then freeze-dried.

##### 3.2.2.1. Phase-Contrast Optical Microscopy

The MBAX microspheres shape and size were estimated using an optical microscope (Olympus-BX51, Olympus American Inc., Center Valley, PA, USA). The particle size of MBAX microspheres was determined by a stage micrometer having an accuracy of 0.01 mm. The average sizes of 100 MBAX microspheres were registered. Pictures in dark-field were taken and the microspheres size distribution was analyzed according to a reference scale.

##### 3.2.2.2. Swelling and Structural Parameters

After gelation, the MBAX microspheres were recovered by filtration, placed in glass vials and weighted. The MBAX microspheres were allowed to swell as described elsewhere [17]. The equilibrium swelling was reached when the weight of the samples changed by no more than 3% (0.06 g). The swelling ratio (q) was calculated by Equation (1):

q = (W_s_ − W_MBAX_)/W_MBAX_ (1)

where W_s_ is the weight of swollen gels and W_MBAX_ is the weight of MBAX in the gel. From swelling measurements, the molecular weight between two cross-links (M_c_), the cross-linking density (ρ_c_) and the mesh size (ξ) values of the different MBAX gels were obtained as reported elsewhere [17]. M_c_, ρ_c_, and ξ were calculated using the model of Flory and Rehner (1943) [37] modified by Peppas and Merrill [38] for gels where the cross-links are introduced in solution 

To determine the nature of water diffusion into the microsphere, the following equation was used [37]:

M_t_/M_o_ = *k* t*^n^* (2)

where M_t_ is the weight of MBAX microspheres at time t, M_o_ is the initial weight of MBAX microspheres, *k* is the kinetic constant, and *n* is the dissolution exponent that characterizing the system. A value *n* < 0.45 indicate a Fickian diffusion mechanism, while 0.45 < *n* < 1 indicate that a non Fickian or anomalous mechanism [39].

##### 3.2.2.3. Scanning Electron Microscopy (SEM)

The surface morphology and microstructure of the freeze-dried MBAX microspheres was studied by field emission scanning electron microscopy (JEOL JSM-7401F, Peabody, MA, USA) without coating at low voltage (1.8 kV). SEM images were obtained in secondary and backscattered electrons image mode.

##### 3.2.2.4. Textural Analysis

The textural analysis was conducted by adsorption/desorption of nitrogen. Surface area was determined by using nitrogen adsorption at their condensation temperature (77.35 °K) and at a relative pressure (*p/p*_0_) of 0.5–0.22 by the BET method [40]. The complete isotherm was conducted at *p/p*_0_ = 0.5–0.99 for adsorption and *p/p*_0_ = 0.995–0.05 for desorption. A surface characterization Autosorb-1 (Quantachrome Instruments, Boynton Beach, FL, USA) was used. The surface of the samples was cleaned at 100 °C for 2 h under vacuum.

#### 3.2.3. Statistical Analysis

All measurements were made in triplicate and the coefficients of variation were lower than 8%. Results are expressed as mean values.

## 4. Conclusions

This study demonstrated that spherical and non-aggregated MBAX microspheres with an average diameter of 531 µm and an average mesh size of 52 nm can be prepared by enzymatic cross-linking. The MBAX microspheres present a porous structure featuring a heterogeneous organization. These structural and textural features confer to MBAX microspheres a high potential for application as microencapsulation systems for bioactive compounds. 
